# Supplementary material for: A hybrid CNN and ensemble model for COVID-19 lung infection detection on chest CT scans
Source: PLoS One. 2023 Mar 9;18(3):e0282608. doi: 10.1371/journal.pone.0282608 (PMC9997961; doi:10.1371/journal.pone.0282608)
Supplement: Attachment — Submitted filename: RL for PLOS One R1.docx [file pone.0282608.s001.docx]

**PLOS ONE**

REPLY TO COMMENTS

**Ref. No.:** PONE-D-21-38730

**Paper Title:** A Hybrid CNN and Random Forest Model for COVID-19 Lung Infection Detection on Chest CT Scans.

Dear Editors and Reviewers,

Thank you for your useful comments and feedback on our paper, which helped us improve its presentation and quality. We have carefully addressed all of your comments in the revised manuscript. We hope that you will be satisfied with the response provided by us.

Sincerely,

The authors.

**Editor's comments**

**Comment 1:** Authors are requested to further enhance the proposed method with more techniques.

**Response 1:** The authors are thankful to the Editor for this comment in the revised version of the manuscript. We extended the paper's contributions by including a comparison between the strong classifier and the weak classifier (i.e., ensemble learning). Besides, we investigated the effect of combing the features extracted using a DL model with the SIFT model, a classic image descriptor. The list of contributions in the revised manuscript is as follows:

1. We proposed comparing deep transfer learning models' performance against using a hybrid classification method. In the proposed hybrid classification method, the features are extracted using a proposed DL model. Then a classic machine learning classifier classifies the chest CT scan images of the infected lung with COVID-19 and normal lung images.
2. We proposed utilizing ensemble learning (e.g., boosting and bagging methods) and a strong classifier (i.e., RF) for image classification.
3. We proposed a new CNN model for feature extraction of COVID-19 lung CT scan images, which is trained from scratch. Then, we proposed extracting the features using the well-known deep transfer learning models. The experimental results show that the quality of the extracted features of the proposed CNN model is better than those features extracted by the well-known deep transfer learning models.
4. The proposed methods are evaluated on five different metrics on one of the most used datasets. The obtained results show that the proposed methods outperformed all existing state-of-the-art methods. Besides, the ensemble learning-based model slightly outperformed the single classifier.

**Comment 2:** Comparison against the state-of-the-arts should be done.

**Response 2:** The authors are thankful to the Editor for this comment. The original manuscript compares the proposed method against the top performing 12 state-of-the-art methods, as listed in Table 2.

**Reviewer #1's comments**

The paper proposes a comparison between two approaches for predicting COVID-19 patients based on the CT scan images. The first approach uses deep transfer learning to classify the images, the second one is based on CNN to extract features from the images and classify them using ramdom forest algorithm.

**Comment:** The paper is well written and easy to understand. The problems being addressed are interesting.

**Response:** The authors are thankful to the Editor for this comment.

**Comment 1:** However, the paper lacked novelty. The authors used deep learning techniques that are well known and widely used in medical image classification.

**Response 1:** The authors are thankful to the Editor for this comment. The main contribution of the proposed work was to answer the research question that whether to use the deep transfer learning, which is the current mainstream, or to design and train a proposed CNN model from scratch for the sake of detecting infected lungs with COVID-19 based on the CT scan images for feature extraction purpose only. The question is raised because the pre-trained models are trained on several types of images (e.g., car, building, and lung scan images.). In contrast, training a DL classifier model from scratch focuses on training the DL model on only lung CT scan images with normal and COVID-19 labels.

Thus, the novelty of this work is two-fold. First the proposed DL model, Fig. 2, for feature extraction. Second, answer the research mentioned above question. Besides, the proposed methods achieved the highest reported results for the widely used dataset.

**Comment 2:** I invite the authors to use more innovative methods and present in-depth the technical details.

**Response 2:** The authors are thankful to the Editor for this comment. As suggested, we extended the paper's contributions by including a comparison between the strong classifier and the weak classifier (i.e., ensemble learning). Besides, we investigated the effect of combing the features extracted using a DL model with the SIFT model, a classic image descriptor. Kindly check the list of contributions on the 2 and 3 pages at the end of the Introduction section.
