# Supplementary material for: A hybrid CNN and ensemble model for COVID-19 lung infection detection on chest CT scans
Source: PLoS One. 2023 Mar 9;18(3):e0282608. doi: 10.1371/journal.pone.0282608 (PMC9997961; doi:10.1371/journal.pone.0282608)
Supplement: Attachment — Submitted filename: AKL_POne-MR response letter.docx [file pone.0282608.s002.docx]

**PLOS ONE**

REPLY TO COMMENTS Ref. No.: PONE-D-21-38730R1

Paper Title: A Hybrid CNN and Random Forest Model for COVID-19 Lung Infection Detection on Chest CT Scans.

Dear Editors and Reviewers,

Thank you for your useful comments and feedback on our paper which helped us to improve its presentation and quality. We have carefully addressed all of your comments in the revised manuscript. We hope that you will be satisfied with the response provided by us.

Sincerely,

The authors.

**Reviewer #2 ‘s comments**

**Comments 2.1:** In the introduction, introduce the problem, motivate the problem, and summarize the contributions
**Response 2.1: The authors are thankful to the reviewer for this comment. As suggested, the revised manuscript includes a modified introduction section with the requested items.**

**Comments 2.2A:** In literature survey, instead of summarizing the works, suggested to discuss the advantages and limitations

**Response 2.2A: The authors are thankful to the reviewer for this comment. As suggested, the revised manuscript includes an improved version of the literature survey. We discussed the main advantages and limitations of the previous methods used to classify COVID-19 by using CT-Scan images.**

**Comment 2.2B:** Authors are suggested to add detailed literature survey and some recommended and related works are

   [EDITORS HAVE REMOVED TO ENSURE BLINDNESS]

**Response 2.2B: The authors are thankful to the reviewer for this comment. As suggested, the revised manuscript increased the literature while the suggested works can’t be accessed by the authors.**

**Comments 2.3:** There are some typos and grammatical mistakes, one such typo is
Because we are dealing with a small dataset, we encouraged to use Scale-Invariant Feature Transform (SIFT) [?]

**Response 2.3: We apologize for such a mistake. In the revised manuscript, we addressed this typo, and we checked for other typos and fix them all.**


**Comments 2.4:** Add more details about the proposed architecture

**Response 2.4: The authors are thankful to the reviewer for this comment. As suggested, we added more details about the proposed two-stage framework. We explained how the features are extracted during the feature extraction phase and how the classification process has been done.**

**Comments 2.5:** Discuss the advantages and limitations of the proposed method
**Response 2.5: The authors are thankful to the reviewer for this comment. As suggested, we updated the list of contributions in the introduction section of the revised manuscript to outline the merits of this work. The limitations of the proposed work are exposed in discussion section (i.e., Section 5.2).**

**Comments 2.6:** Compared the proposed approach with at least recently published 3 methods
**Response 2.6: The authors are thankful to the reviewer for this comment. In the initial submission, we already compared the proposed methods against 13 recent methods in Table 3.**

**Comments 2.7:** Add the level of trust of the proposed method

**Response 2.6: The authors are thankful to the reviewer for this comment. As suggested, the revised manuscript includes the examination of the cross-validation which outlines the variance of the proposed method.**

**Reviewer #3’s comments**

**Comment:** This paper presents a new CNN architecture for feature extraction and then to use the random forest (RF) for COVID-19 diagnosis.

The paper is well written and the presentation is adequate. However, the quality of the paper should be improved in parts.

**Response: Thank you!**

**Comment 3.1:** The author claims in the abstract that "To our knowledge, there is no research conducted to investigate whether to use the transfer deep learning models CT scan image classification against feature extraction."
I totally disagree on this since the following book chapter uses transfer learning models for deep feature extraction and classical ML methods for classification.
Abdulhamit Subasi, Arka Mitra, Fatih Ozyurt, Turker Tuncer, Automated Covid-19 detection from CT images us-ing deep learning in Editor(s): Varun Bajaj, G R Sinha, Computer-aided Diagnosis and Design Methods for Biomedical Applications, CRC Press, Taylor & Francis, 2021.

**Response 3.1: The authors are thankful to the reviewer for this comment. As suggested, we modified this sentence to be more accurate. Abdulhamit Subasi et al. examined the quality of the extracted features from DL models by classifying these extracted features with the help of different ML models. The demerits of this work are three-fold. First, the authors utilized a small dataset, 349 CT scan images for the COVID-19 class and 397 CT scan images for the non-COVID19 class. Second, the authors did not explore the training from the scratch approach for feature extraction of COVID-19 classification datasets. Third, the authors did not compare their proposed methods with the state-of-the-art methods. Moreover, we studied comparing DL models only for feature extracting methods against DL with other feature extraction methods used with ML models such as SIFT. We found that using DL only for feature extraction can be improved by fusing the extracted features of DL models and SIFT image descriptors, as listed in Table 2 in the revised manuscript. Moreover, Table 2 shows that using the proposed DL architecture performs better than the transfer deep learning models, as listed in Table 3 in the revised manuscript.**

**Comment 3.2:** The novelty they claimed is not enough. Mayme they may say that their CNN model created from scratch achieved good result.

**Response 3.2: The authors are thankful to the reviewer for this comment. As suggested, we explained the novelty of our work in the list of contributions in the introduction section of the revised manuscript. The second contribution covers the suggested point by the reviewer.**

**Comment 3.3A:** The title "A Hybrid CNN and Random Forest Model for COVID-19 Lung Infection Detection on Chest CT Scans" should be changed since it says Random forest, but RF did not achieved the best results after the revision, ensemble SVM achieved best accuracy.

Hence The title might be "A Hybrid CNN and Ensemble Model for COVID-19 Lung Infection Detection on Chest CT Scans"

**Response 3.3A: The authors are thankful to the reviewer for this comment. As suggested, the title was modified.**

**Comment 3.3B:** What is the significant advantage of the proposed method against previously published ones?

**Response 3.3B: The authors are thankful to the reviewer for this question. We updated the list of contributions in the introduction section of the revised manuscript to answer this question. We can summarize the main contribution of our work as follows:**

1. ***We proposed studying the performance of DL as an end-to-end classification model, i.e. deep transfer learning, for CT scans of COVID-19 against using DL models only for feature extraction and then using an ML model for the purpose of image classification.***
2. ***We proposed a new CNN architecture that is trained from scratch. Then, we compared the quality of the extracted features from CT scan images using the well-known DL models (e.g., VGG and MobileNet) against the proposed CNN architecture. The results showed that the proposed CNN architecture extracted features led to higher classification accuracy rates.***
3. ***We compared the results of fusing the extracted features from DL models with the SIFT image descriptor, which resulted in improving the classification accuracy rate.***
4. ***We compared the performance of the classic ML methods against the ensemble learning. The results showed that the ensemble learning models slightly outperformed the classic ML methods.***

**Comment 3.4:** The literature review is not comprehensive. Authors are suggested to review more new and relevant research to support their research contribution.

**Response 3.4: The authors are thankful to the reviewer for this comment. As suggested, we improved the literature review by adding more recent papers for COVID-19 classification. Besides, we mentioned the main advantages and limitations of the previous research methods.**
