# Supplementary material for: A hybrid CNN and ensemble model for COVID-19 lung infection detection on chest CT scans
Source: PLoS One. 2023 Mar 9;18(3):e0282608. doi: 10.1371/journal.pone.0282608 (PMC9997961; doi:10.1371/journal.pone.0282608)
Supplement: Attachment — Submitted filename: RL.docx [file pone.0282608.s003.docx]

**PLOS ONE**

REPLY TO COMMENTS Ref. No.: PONE-D-21-38730R2

Paper Title: A Hybrid CNN and Random Forest Model for COVID-19 Lung Infection Detection on Chest CT Scans.

Dear Editors and Reviewers,

Thank you for your useful comments and feedback on our paper which helped us to improve its presentation and quality. We have carefully addressed all of your comments in the revised manuscript. We hope that you will be satisfied with the response provided by us.

Sincerely,

The authors.

**Reviewer #4’s comments:**

**Comment 4.1.** What is the robustness of the proposed method?

**Response 4.1:** The authors are thankful to the reviewer for this comment. As suggested, the revised manuscript includes the examination of the cross-validation technique to test the model's robustness regarding data change (i.e., testing the model accuracy with different training datasets). In addition, we computed the confidence interval (CI) with a confidence level of 95% for the 10 folds and provided a figure that depicts the results. The obtained CI show that the model is robust as the change in the proposed model accuracy is very tiny due to changing the training data.

2. Write about the advantages of the suggested method over other existing methods?

**Response 4.2:** The authors are thankful to the reviewer for this question. We updated the list of contributions in the introduction section of the revised manuscript to answer this question. We can summarize the main contributions of our work as follows:

1. We proposed studying the performance of DL as an end-to-end classification model, i.e., deep transfer learning, for CT scans of COVID-19 against using DL models only for feature extraction and then using an ML model for the purpose of image classification.
2. We proposed a new CNN architecture that is trained from scratch. Then, we compared the quality of the extracted features from CT scan images using the well-known DL models (e.g., VGG and MobileNet) against the proposed CNN architecture. The results showed that the proposed CNN architecture extracted features led to higher classification accuracy rates.
3. We compared the results of fusing the extracted features from DL models with the SIFT image descriptor, which resulted in improving the classification accuracy rate. To our knowledge, this is the first study of fusing features extracted by the SIFT method and DL models.
4. We compared the performance of the classic ML methods against the ensemble learning models. The results showed that the ensemble learning models slightly outperformed the classic ML methods. To our knowledge, this is the first comparison between these two types of methods on this dataset.
5. The proposed methods are evaluated on five different metrics on one of the most used dataset. The obtained results show that the proposed methods outperformed all of the existing state-of-the-art methods. Besides, the ensemble learning-based model slightly outperformed the single classifier.

3. The originality of the paper needs to be stated clearly.

**Response 4.2:** The authors are thankful to the reviewer for this question. The revised version of the manuscript includes the novelty in the list of contributions in the introduction section as follows:

1. We compared the results of fusing the extracted features from DL models with the SIFT image descriptor, which resulted in improving the classification accuracy rate. To our knowledge, this is the first study of fusing features extracted by the SIFT method and DL models.
2. We compared the performance of the classic ML methods against the ensemble learning models. The results showed that the ensemble learning models slightly outperformed the classic ML methods. To our knowledge, this is the first comparison between these two types of methods on this dataset.
